# Next-Generation Community Air Quality Sensors for Identifying Air Pollution Episodes

**DOI:** 10.3390/ijerph16183268

**Published:** 2019-09-05

**Authors:** Edmund Seto, Graeme Carvlin, Elena Austin, Jeffry Shirai, Esther Bejarano, Humberto Lugo, Luis Olmedo, Astrid Calderas, Michael Jerrett, Galatea King, Dan Meltzer, Alexa Wilkie, Michelle Wong, Paul English

**Affiliations:** 1 Department of Environmental and Occupational Health Sciences, School of Public Health, University of Washington, Seattle, WA 98195, USA; 2 Comite Civico del Valle, Brawley, CA 92227, USA; 3 Study Community Steering Committee Member, Brawley, CA 92227, USA; 4 Department of Environmental Health Sciences, School of Public Health, University of California, Los Angeles, CA 90095, USA; 5 Public Health Institute, Oakland, CA 94607, USA; 6 California Department of Public Health, Richmond, CA 94804, USA

**Keywords:** air quality, sensors, community-engaged research, community-based participatory research, citizen science

## Abstract

Conventional regulatory air quality monitoring sites tend to be sparsely located. The availability of lower-cost air pollution sensors, however, allows for their use in spatially dense community monitoring networks, which can be operated by various stakeholders, including concerned residents, organizations, academics, or government agencies. Networks of many community monitors have the potential to fill the spatial gaps between existing government-operated monitoring sites. One potential benefit of finer scale monitoring might be the ability to discern elevated air pollution episodes in locations that have not been identified by government-operated monitoring sites, which might improve public health warnings for populations sensitive to high levels of air pollution. In the Imperial Air study, a large network of low-cost particle monitors was deployed in the Imperial Valley in Southeastern California. Data from the new monitors is validated against regulatory air monitoring. Neighborhood-level air pollution episodes, which are defined as periods in which the PM_2.5_ (airborne particles with sizes less than 2.5 μm in diameter) hourly average concentration is equal to or greater than 35 μg m^−3^, are identified and corroborate with other sites in the network and against the small number of government monitors in the region. During the period from October 2016 to February 2017, a total of 116 episodes were identified among six government monitors in the study region; however, more than 10 times as many episodes are identified among the 38 community air monitors. Of the 1426 episodes identified by the community sensors, 723 (51%) were not observed by the government monitors. These findings suggest that the dense network of community air monitors could be useful for addressing current limitations in the spatial coverage of government air monitoring to provide real-time warnings of high pollution episodes to vulnerable populations.

## 1. Introduction

As part of a community-engaged research study aimed at addressing the high levels of PM_2.5_ air pollution (airborne particles with sizes less than 2.5 μm in diameter) and high rates of asthma hospitalizations in the Imperial Valley, California, PM air quality monitors were deployed over a two-year period from 2015–2016. By spring 2017, 38 community air monitors were installed at permanent locations, supplementing the existing network of six government PM_2.5_ monitoring sites in the area. The overarching aim of this study and the community-engagement process is to fill the need for more spatially and temporally refined and real-time data on PM levels in the Imperial Valley, a region that often exceeds air quality standards [1,2].

This study involves various air quality stakeholders with complementary roles [2]. The California Environmental Health Tracking Program led the community engagement process by working with our community partner to form the community steering committee (CSC), which is made up of local environmental leaders and residents to help guide the project. By working with the CSC in a series of meetings, community needs and vulnerable areas of the valley are identified and potential sites are selected for new community air monitors. Researchers at the University of Washington developed new air monitors based on so-called next-generation sensor technology [3,4,5], specifically, light-scattering optical particle counters that are lower in cost than the conventional US Environmental Protection Agency (US EPA) federal reference method (FRM) and federal equivalent method (FEM) particle mass measurement instruments. The monitors were developed, tested, calibrated by the researchers, and validated in collaboration with the state air quality agency, the California Air Resources Board (CARB) [6]. A researcher at the University of California, Los Angeles serves as a scientific advisor for the project and helps to educate community members about the health effects of air pollution. The main community partner in the study is a local environmental justice organization, Comite Civico del Valle (CCV), which helps the team identify where new air quality information will help to protect the health of vulnerable populations. Additionally, they also help to deploy and maintain the monitoring network.

An important aspect of next-generation air monitoring is the ability of the new technology to provide the public with more immediate and relevant information [3,4,5]. Often, real-time data are provided via websites and services that can warn individuals when air pollution levels are above thresholds, and thus are no longer deemed safe for human health. In Imperial, school children are taught to follow the US EPA’s AirNow asthma flag program, in which different color flags are displayed at schools to indicate to asthmatic children whether it is safe to exercise outdoors [7]. Consequently, community partners asked whether the new community monitors could provide timely information on the presence of elevated air pollution episodes so that vulnerable individuals may take action to reduce their exposures to PM_2.5_.

In this paper, we assess the ability of the Imperial community air monitors to identify air pollution episodes. We define air pollution episodes as periods during which hourly PM_2.5_ concentrations exceed 35 μg m^−3^, the 24-h level under the National Ambient Air Quality Standards (NAAQS). We then compare the number and location of episodes observed by either the government or the community air quality monitoring networks, and the number of times in which community air monitors identify episodes that were not observed by the government monitors.

## 2. Materials and Methods

### 2.1. Study Area

The Imperial Valley is located in Imperial County in California along the US–Mexico Border. The valley consists of a number of small cities and towns mostly surrounded by agricultural land and open desert space. According to the most recent census estimates, approximately 180,000 people live in the county, with more than 80% of residents being of Hispanic origin [8]. The geography of the valley is largely desert landscape, except for the heart of the valley that has been converted for agricultural use. The Salton Sea is at the Northern end of the valley, which is undergoing large environmental changes due to water resource policies that have resulted in a receding water line and large areas of exposed dry shoreline, which may be a new source of airborne particulate matter. The city of Mexicali, Mexico, with over 680,000 residents [9], is located at the Southern end of the valley. The border area near Mexicali experiences heavy and congested traffic; cross-border transport of urban air pollution is a concern for Imperial residents on the US side of the border. Previous research has demonstrated that cross-border air pollution follows the prevailing northwesterly wind flow [10]. The continued growth and industrialization of Mexicali adds to the air pollution of the region. The valley contains a number of potential PM sources, including dust from the desert and Salton Sea beds, as well as local, port of entry, and cross-border mobile emissions, agricultural dust and burning, and industrial activities on both sides of the border.

### 2.2. Government Air Monitoring Network

**Figure 1:**
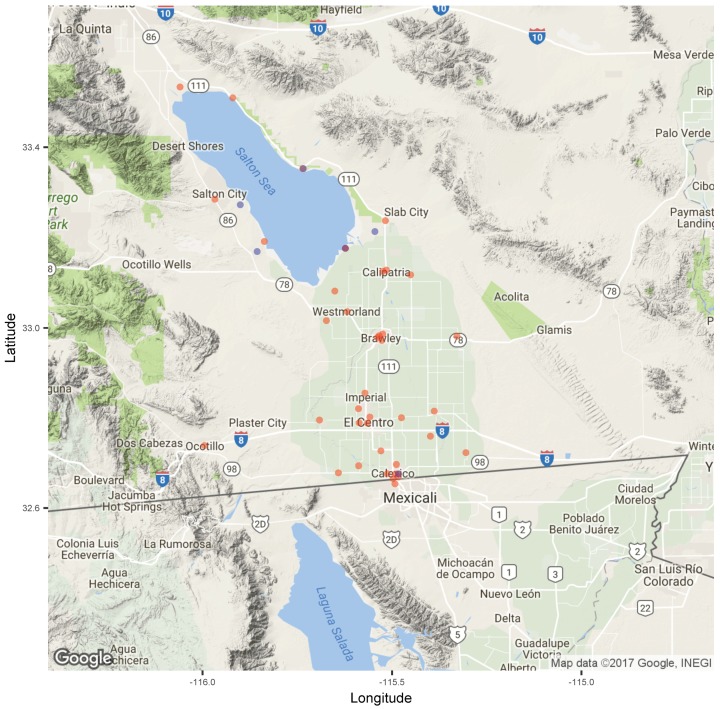
Map of the government and community air quality monitors, with government-operated sites shown as blue dots, and community monitoring sites in red. Purple dots indicate sites with colocated government and community monitoring.

Hourly PM_2.5_ data for the government-operated monitors were obtained from the CARB for the study period from 1 October 2016 to 28 February 2017 [11]. During this period, six sites monitored PM_2.5_ (Figure 1). Some of the sites collected measurements using the beta attenuation monitor (BAM) instrument, while others used the tapered element oscillating microbalance (TEOM) instrument, but all reported data to the CARB, and only the data observations that met CARB Quality Assurance and Quality Control (QA/QC) [12] (145 observations were marked as “suspect” in CARB’s QA/QC, and were not included in the analysis) were included in the analysis (1405D TEOM Thermo Fisher Scientific, Franklin, MA operated at Bombay Beach, Naval Test Base, Salton City, and Sonny Bono sites; 1020 BAM Met One, Grants Pass, OR, operated at Calexico–Ethel Street and Niland–English Road sites). Five of the six sites were located around the Salton Sea. The remaining government-operated sites were located in Calexico, the Southern-most city in the valley on the US side of the border, adjacent to Mexicali. No hourly government PM_2.5_ monitoring existed in the towns between Calexico and Niland, which include the cities of El Centro (population 42,596), Imperial (14,752), and Brawley (24,953). However, daily PM_2.5_ data were available for Calexico–Ethel, Brawley, and El Centro-9th sites. Currently, 1-h ambient air quality standards do not exist for the NAAQS or for the California Ambient Air Quality Standards. For our analysis, we defined any hour at or above a concentration of 35 μg m^−3^ as an “elevated air pollution hour”. This concentration level corresponds to the 24-h NAAQS. Furthermore, we defined an “air pollution episode” as a period of one or more consecutive hour made up of elevated air pollution hours. For example, measurements at a site for two consecutive hours (e.g., 09:00 and 10:00) with concentrations of 50 and 51 μg m^−3^ would be considered to be two elevated air pollution hours, contributing to a single air pollution episode. Finally, because our definition of an episode allowed it to last a variable number of hours, and episodes could have overlapped and be double counted across both government and community monitoring sites, we defined a separate metric, an “episode day”, as a day in which any of the hours were equal to our greater than 35 μg m^−3^. This allowed us to compare the number of days during the study period that were similarly and dissimilarly identified as an episode day. Ozone and NO_2_ were also monitored in the valley. During the study period, the mean hourly O_3_ concentration at the Calexico–Ethel site was 0.026 ppm, and none of the sites reported concentrations above the 1-h California ozone standard (0.090 ppm). The average NO_2_ concentration during the study period was 0.014 ppm at the Calexico–Ethel site, and none of the sites reported concentrations above the 1-h California NO_2_ standard (0.180 ppm).

### 2.3. Community Air Monitoring Network

Over the two years leading up to the study period, 40 community air quality monitors were installed in vulnerable areas in the valley. A community-engaged process was used to identify the locations for these sites [2]. Briefly, 11 neighborhoods were prioritized for monitoring by the CSC, a principal components analysis of land use factors in each of these neighborhoods was conducted to identify areas within the neighborhoods that represent specific combinations of land uses, community residents selected potential sites from these areas, and CCV obtained permission from the property owners of the sites and installed the monitors.

Each community monitor included a low-cost PM sensor, a Dylos 1700 (Dylos Corporation, Riverside, CA) laser particle counter, which was modified to measure PM in four size bins (>0.5, >1, >2.5, and >10 µm). The Dylos was connected to a custom circuit board developed at the University of Washington, which saved the data over a wireless Internet connection to data servers hosted at the University. The system also included measurements of temperature and relative humidity, which were used to calibrate particle counts measured by the four-bin Dylos to FEM BAM and FRM mass measurements operated by the CARB [6]. This enabled the conversion of the counts to mass concentrations. The sensor and associated electronics were housed within a protective enclosure, which had a manifold designed to bring outside air directly to the sensors for measurement, with little residence time in the enclosure. All monitors operated on wall power and were installed on rooftops or unobstructed sides of buildings at each site. The cost of each monitor (in parts, but not including labor to assemble monitors) was approximately $1500 USD.

Two of the monitoring sites were installed late in the study period, and thus missed most of the data for this study and were excluded. This left 38 sites with data for analysis. For these remaining sites, hourly average PM_2.5_ levels were converted to mass concentrations based on an hourly FEM BAM calibration relationship developed by our group and the CARB [6]. The equation for the sensor calibration is provided in Supplementary Information, with further details provided in our previous sensor calibration paper [6]. The previous study found that the calibration produced positively correlated mass concentrations at the hourly scale against an FEM BAM (R^2^ = 0.79), and was comparable to the correlation observed in that study between government-operated colocated FEM and FRM instruments (R^2^ = 0.73). Over the six-month colocation period in that study, the calibrated community monitor reported an identical concentration (12.6 μg m^−3^) to the FEM BAM. The calibrated community monitor was also compared to a colocated Portable Environmental Beta-Attenuation Monitor (E-BAM) at the same site and reported calibrated concentrations within 3% of each other. A 35 μg m^−3^ threshold was used to identify elevated air pollution hours and episodes for each monitor. The proportion of elevated hours in the total monitoring hours was compared between government versus community monitoring, as well as the proportion of episodes per monitoring hour using the chi-square test. Additionally, the paired comparison of days observed to be an episode day by the government versus community monitoring was assessed using the McNemar’s test. All analyses were conducted in R 3.1.0 (The R Foundation for Statistical Computing).

## 3. Results

### 3.1. PM_2.5_ Episodes from Government Monitoring

**Figure 2:**
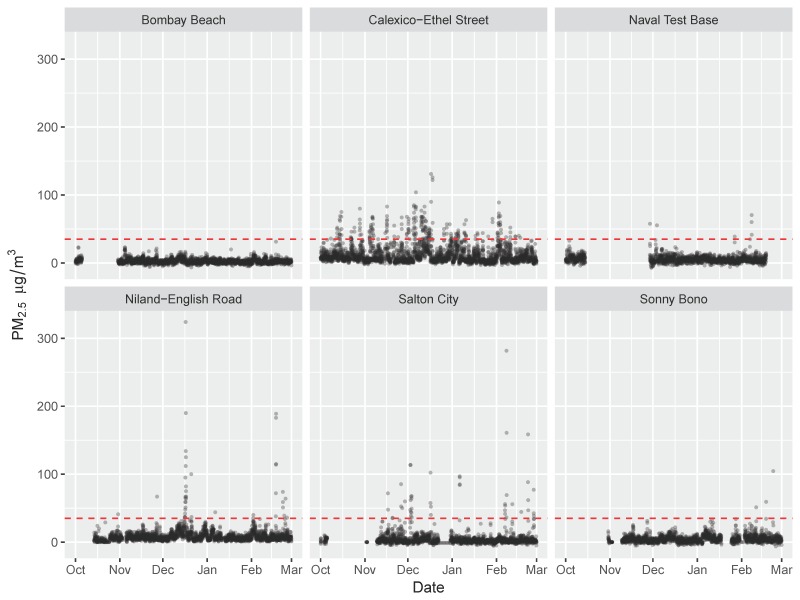
Time-series of hourly PM_2.5_ (airborne particles with sizes less than 2.5 μm in diameter) data reported for each of the government monitoring sites over the study period.

Figure 2 illustrates the time-series of data collected at each of the government monitoring sites over the study period. Hourly PM_2.5_ was not complete for any of the government monitoring sites. As only a small proportion of the data (0.9%) were excluded due to CARB’s QA/QC flags, the incomplete data are due to missing data or no monitoring being done at those times. Data were mostly complete for the Calexico–Ethel Street site (3499 hourly observations out of a possible 3624 h in the study period, or rather, 97% complete), which also had the highest mean hourly concentration, the highest number of hours at or above 35 μg m^−3^, and the greatest number of episodes (Table 1). A density plot of concentrations at each site, with concentrations above 35 μg m^−3^ is provided in Supplementary Information (Figure S1). At this site, most of the episodes occurred at midnight, but quite a few episodes also occurred between the hours of 04:00–07:00 and after 18:00, and most episodes occurred on Saturdays (Figures S2 and S3). Calexico–Ethel Street is the Southern-most government monitoring site, located near the US–Mexico border. In contrast, the Niland–English Road site, located in the Northern part of the valley, just South of the Salton Sea, recorded an average concentration that was 74% lower than the average for Calexico–Ethel. Moreover, only 16 air pollution episodes were observed at Niland–English, compared to 77 episodes observed at Calexico–Ethel. Most of the episodes were associated with winds from the West (Figure S4).

Across all government sites, 116 episodes were observed. Of these, 104 were unique, in that only one government site identified elevated levels of PM_2.5_ during that time. Conversely, 12 of the episodes overlapped in time between multiple sites. For those sites that overlapped, most of the time (10 of the 12), only one other government monitoring site identified the same episode. Thus, there was little spatial overlap in the government monitoring network in terms of identifying PM episodes.

### 3.2. PM_2.5_ Episodes from Community Monitoring

**Figure 3:**
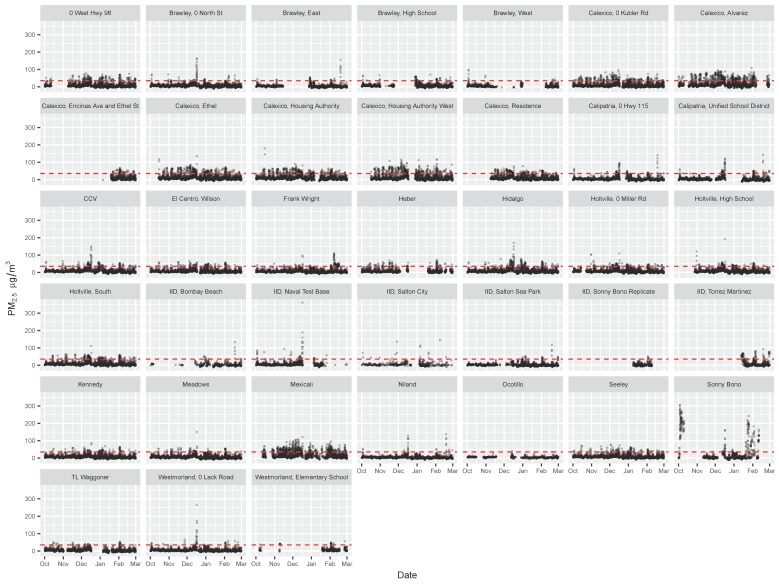
Time series of hourly PM_2.5_ data reported for each of the community monitoring sites over the study period.

Time-series plot and summary statistics for each of the community monitoring sites are provided in Figure 3 and Table 2. Density plots of concentrations observed at each site are provided in Figure S5. Eight of the community sites had data that were at least as complete as the most complete Calexico–Ethel government site (>97% data complete), while nine community sites had data that were less complete than the least complete Naval Test Base government site (<59% complete). The QA/QC flags provided for the community monitoring data indicated that 4% were excluded from analysis due to monitor malfunction, and 7% were excluded due to likely network connection issues. The Calexico–Ethel community site was a colocation site with the Calexico–Ethel government site. Similarly high numbers of hourly observations were collected from both government and community monitoring instruments, and the average concentrations were similar (12.13 and 13.51 μg m^−3^, respectively).

The highest average concentrations observed by the community monitoring network were considerably higher than those observed by the government monitoring network. Notably, a high average concentration of over 20 μg m^−3^ was observed at the Mexicali community site, which is located on the Mexican side of the border. Additionally, the Calexico Alvarez community site, which is close to the border crossing, measured a higher average concentration than the Calexico–Ethel community site, which is farther away from the border (18.56 vs. 13.51 μg m^−3^, respectively), even though both are situated within the same town of Calexico. In the Northern part of the valley, the highest average concentration, 27.18 μg m^−3^, was observed at the Sonny Bono site, which is close to the Salton Sea. This concentration was considerably higher than the concentration measured by government instruments at the Sonny Bono site—however, both the government data as well as the community monitoring data were fairly incomplete, and the collected data do not perfectly align with each other in time.

The proportion of elevated air pollution hours to total monitoring hours was greater for community monitoring compared to government monitoring (5.41 vs. 2.03 per 100 monitoring hours, respectively, *p* < 0.001). In terms of air pollution episodes (i.e., one or more contiguous elevated hours), more than 10 times as many PM_2.5_ episodes were identified by the community monitoring network than the government monitoring network (1426 vs. 116 episodes). At our Calexico–Ethel colocation site, the number of episodes identified by the community monitor and the government-operated monitor were not significantly different (83 vs. 77, respectively, *p* = 0.32), which was expected for the two types of monitors located within a few meters of each other. Additionally, a high number of episodes were observed at the Mexicali and Calexico Alvarez community sites, which was consistent with the higher average concentrations that were observed at these two sites in general. The proportion of episodes to total monitoring hours was greater for community monitoring compared to government monitoring (14.5 vs. 7.02 per 1000 monitoring hours, respectively, *p* < 0.001).

Similar to the government monitoring sites, some of the episodes observed at the community sites might have overlapped in time with episodes observed at other sites in the network. Perhaps due to the higher spatial density of the community monitors compared to government monitoring, the majority of episodes observed tended to be shared across sites (i.e., when an episode was observed at one community site, at least one other community monitoring site observed it). Out of the 1426 episodes, only 122 were unique to a single site. Of the episodes that overlapped, most (964 out of 1304) overlapped with at least five other community sites, which suggested that the network could identify regional high PM episodes that affect multiple monitors and populations in multiple neighborhoods.

### 3.3. To What Extent Did Community Monitoring Identify Episodes Missed by Government Monitoring?

While the aforementioned results suggest that community monitoring identified more PM_2.5_ episodes than government monitoring and that the majority of episodes observed at community monitoring sites were shared among community sites, for each of the 1426 episodes, we also assessed whether they overlapped with any episodes identified by the government monitoring. Forty-nine-percent (703 of the 1426 episodes) corresponded to episodes that were also detected by the government monitoring network. The remaining 723 episodes were unique and only detected by the community monitoring network.

In a paired comparison of episode days (i.e., days in which PM_2.5_ levels reached the 35 μg m^−3^ threshold for at least one hour during the 24-h period), of the 79 days that were identified as air pollution episode days by the government monitoring, 74 days (93.7%) were also identified as episode days by the community air monitoring network (Table 3). However, an additional 56 days were identified by the community monitoring network as air pollution episode days that were not identified by the government-operated network. The difference in the proportion was significant (*p* < 0.001). Note that community monitoring did not recognize all government episode days, as five episode days, identified by government monitoring, were not recognized by community monitoring, which suggested that short-term PM episodes might be spatially localized and not necessarily easily to identify across community sites.

## 4. Discussion

There are many potential benefits of engaging community stakeholders to collect richer and more relevant environmental monitoring data to inform environmental policy and planning. Community-engaged research may improve collective knowledge and build consensus towards an understanding of the hazards that exist within a community. Moreover, research that involves community scientists who are familiar with the local population and context for exposure may help to better identify vulnerable populations who are exposed to these hazards. Participation from various stakeholders, including residents, academics, and government representatives, may also promote and demonstrate a “team science” approach towards a collaboratively collecting, interpreting, communicating, and responding to data to affect change. While citizen science is now a well-recognized approach for engaging individuals to collect data with scientific goals in mind, the approach taken in the Imperial Air study was notably different in that three main partners took on different responsibilities in collaboration and engaged in a systematic process to design and develop a new monitoring network with the goal of collecting novel air quality information. Furthermore, various air quality stakeholders participated in the study to ensure that the data collected met goals for data validity, both from the perspective of understanding the performance of the community air monitoring sensors in comparison to FEM and FRM instruments, as well as from the perspective of where monitoring should be conducted to best improve information for the community.

This paper is the first that we are aware of that considers the quantitative benefits of a dense network of community air monitors, that is, in terms of being able to identify air pollution episodes in an air shed. Quantifying these benefits is particularly important because government-operated air quality monitoring already exists in many communities across the US, and some may hold the perception that there would be little added informational value from augmenting government monitoring with community monitoring. However, a strong motivating factor for communities to begin their own air quality monitoring may come about due to either real or perceived problems with access, trust, or understanding of government air quality data, as well as a perception that better monitoring is possible.

The Imperial Valley is arguably a well-monitored region for PM_2.5_ air quality. For a predominantly rural community with less than 200,000 residents and a low population density, it is unusual to find as many as six government-operated hourly PM_2.5_ monitoring sites (and three 24-h PM_2.5_ monitoring sites). Moreover, the spatial arrangement of the monitors captured many of the major air pollution concerns for the region, including potential emissions and transport at the border (measured at the Calexico–Ethel site), and air quality by the Salton Sea (measured at Niland and other sites). However, a challenge with rural areas like Imperial is that there are many small towns with vulnerable populations that are not located near the government monitors. The advent of lower-cost PM instruments has improved the feasibility of collecting monitoring data in these various neighborhoods. In a recent work in which we developed a land use regression model using data from the community monitoring network for different particle size ranges [13], PM_2.5_ and PM_coarse_ (PM_coarse_ refers to particles with sizes between 2.5 and 10 μm in diameter) concentrations were found to vary across the valley, with the concentration of larger particles related to seasonal wind patterns, the desert, and fallow agricultural land use, and smaller particles related to road and urban land use in the models.

In our study, we found that hourly PM_2.5_ concentrations on rare occasions (1% of the time) reached levels as high as 49 μg m^−3^ at the government monitoring sites in the valley during the period from October 2016 to February 2017. Most of the time when this occurs, only one government monitor in the valley detects the episode. Therefore, the community would be highly reliant on continuous high-quality monitoring at all sites to identify episodes and issue public health warnings. However, we found that this is not always the case. Even the best operating government site (Calexico–Ethel) only provided 97% data completeness during the study period.

Comparatively, the community monitoring network did not perform much more reliably than the government network. Approximately, just as many community sites collected more data than the best government site as did sites that collected less data then the worst government site. However, we found that the community network identified more than 10 times as many air pollution episodes than the government network. Moreover, perhaps because of the larger number and spatial density of the community monitors, we found that when an episode was identified by one monitor, it was usually identified by others in the network. From a reliability standpoint, a community might be less reliant on any single community monitor that could fail or produce inaccurate data if other monitors existed in the network that still operate and can corroborate that an air pollution episode is occurring. The probability of identifying local pollutant emission events may be higher by having many monitors in a large spatially distributed network, and the extent of far-field pollutant events might also be better characterized by the use of many monitors.

**Figure 4:**
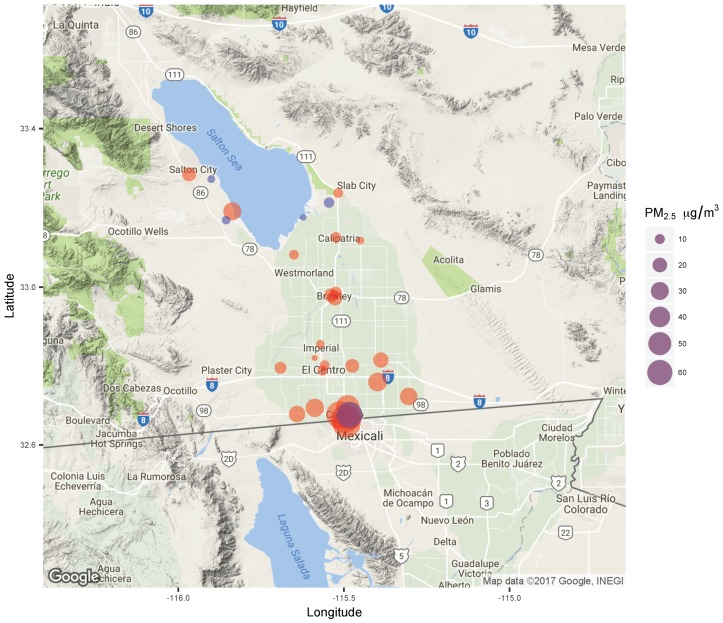
An air quality episode identified by the community network (red points) on 10 December 2016, along the US–Mexico Border, during which average concentrations ≥35 μg m^−3^ were observed at six community sites (five at the Calexico sites and the Mexicali site), as well as at the one government-operated site in the area (Calexico–Ethel in purple). Other government sites are in blue.

**Figure 5:**
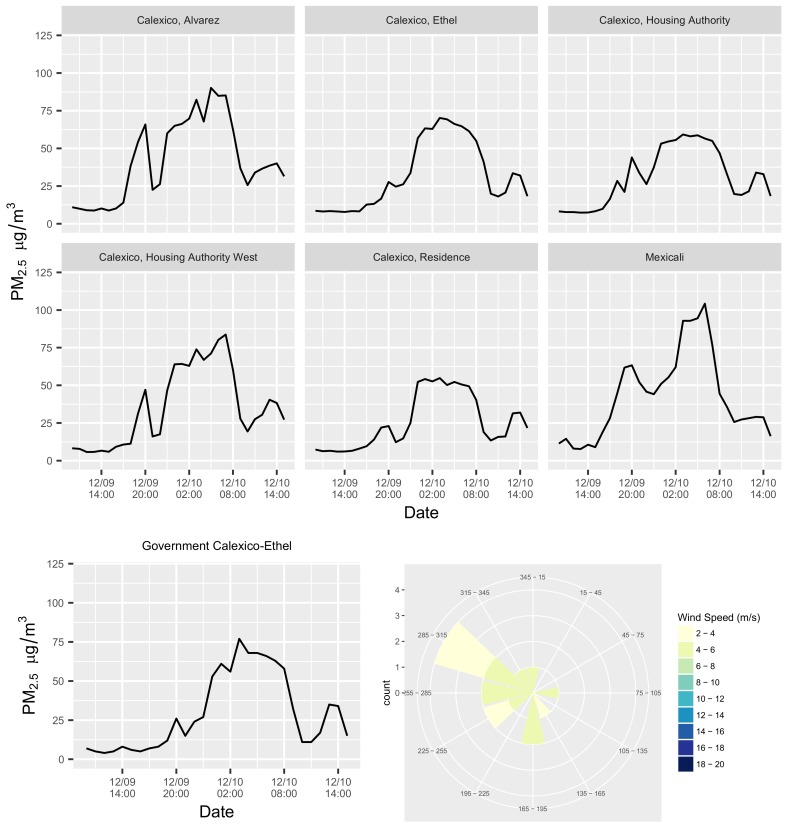
Time-series of concentrations observed during the 10 December 2016 episode at different sites (the government-operated Calexico–Ethel site is on the bottom left). The wind rose during the episode (bottom right).

To illustrate this point, Figure 4 shows one such episode, which occurred on 10 December 2016, during a period in which the wind blew mainly from the west. The episode was detected by the government monitoring site at Calexico–Ethel, as well as by six of the community monitoring sites. During the episode, the time-series of concentrations measured at the colocated community and government monitors at the Calexico–Ethel site were closely matched, and both reached peak concentrations of approximately 75 μg m^−3^ at 3:00 am (Figure 5). However, the highest hourly concentration during the episode was observed at the Mexicali site, later in the morning at 07:00. The time-series of concentrations for different sites demonstrated small-area spatial correlations. Notably, for this episode, there was a slightly smaller and shorter concentration peak that occurred on the evening of the 9 December at 20:00, before the longer and larger concentration peak on 10 December. This was mainly observed at the subset of sites that are closer to the border crossing: Calexico Alvarez, the two Housing Authority sites, and the Mexicali site. The short peak was much less apparent at the Calexico–Ethel colocation site, which is farther from the border. Thus, this one episode provides insights into how data from multiple community and government monitors may be considered collectively to corroborate an air pollution episode, and provides some hints at the spatio-temporal variability of pollution concentrations during the episode.

While promising, limitations exist with respect to current low-cost air monitoring. The measures from the community monitors used in the Imperial study are non-regulatory, and despite our work calibrating and validating the community monitors’ sensor measurements to FEM and FRM instruments in collaboration with regulatory government agencies, the purpose of our community network was not to inform regulatory compliance. However, we have found in this paper that non-regulatory monitoring may still be useful for identifying changing pollutant concentrations that would otherwise not be detected by existing government monitoring. Moreover, as performance targets and criteria are increasing, deliberated, and assessed, concerns over the compatibility of the information collected from regulatory and non-regulatory instruments may become less of an issue [5].

Another limiting factor of regulatory monitoring has to do with siting guidelines that provide requirements for where and how regulatory monitoring will occur. These guidelines were deemed too restrictive for many of the sites that our community partners had identified as important locations to understand local emissions, concentration hotspots, or exposure to vulnerable populations like school children. In this paper, we found that having many monitoring sites can be helpful for identifying air pollution episodes. Moreover, some of our sites would probably have been considerably more difficult for government agencies to establish. For instance, one of the highest concentration sites we observed was the Mexicali site on the Mexican side of the border. However, for our team, this site was only slightly more difficult to implement compared to the other community sites on the US side of the border because of the cooperation and help of the CSC.

It is unclear what impacts these small-area, short term PM_2.5_ episodes have on the health of those exposed in the Imperial Valley. The original motivation for the project was to better inform asthmatics, as there is strong evidence of the association between ambient air pollution and the exacerbation of symptoms for those with pre-existing asthma, as well as a potential linkage to asthma incidence [14]. Short-term exposures to PM_2.5_ have been found to be associated with asthma symptoms in children and adults in two previous California studies [15,16], and more recently in a large US panel study, to be related to digital rescue inhaler use [17]. PM exposure assessed using information from the community air monitoring network are currently being used in a follow-up National Institutes of Health study of asthmatic school-aged children in the Imperial Valley.

An important aspect of next-generation air monitoring is the ability of the new technology to provide the public with more immediate and relevant information [3,4,5]. Real-time data are often provided via websites and services that can warn individuals when air pollution levels are above thresholds that are no longer deemed safe for human health. In Imperial, school children are taught to follow the US EPA’s AirNow asthma flag program [7], and different colored flags are displayed at schools to indicate to asthmatic children whether it is safe to exercise outdoors. A colored-coded flag program can be similarly based on community air monitoring data.

As of July 2019, the Imperial Air community monitoring network is still in operation, and data from the monitoring network are available in real-time on a community website (http://www.ivan-imperial.org/air). Residents can visit the website to access data from their nearest community monitoring site and see updated color-coded public health messages aimed at informing vulnerable individuals in order to reduce their exposure during air pollution episodes. A link is also provided to similar color-coded messages that are released by the local air quality agency based on the government-operated network. Guidance is also provided on the website for individuals about the differences between community vs. government air quality monitoring, as well as a suggestion to follow the more health protective message provided by either monitoring network.

## 5. Conclusions

In the Imperial Valley, a community monitoring network made up of many low-cost PM sensors detected more than 10 times as many air pollution episodes than the relatively small number of government-operated air quality monitors in the region. While many of the episodes identified by the community network were also identified by the government monitors, approximately 50% were episodes that were only identified by the community network. Most of the time, when one community monitor detected an episode, other monitors in the community network also detected the episode. This suggests that there may be value in augmenting the limited spatial coverage of current government-operated air quality monitoring with dense community monitoring to provide a more reliable approach to identifying pollution episodes.

## Figures and Tables

**Table 1:** Site data summary statistics of concentrations and air pollution episodes for government sites during the study period (1 October 2016 to 28 February 2017).

Site	Hourly Observations *	PM_2.5_ Concentration μg m^−3^ Mean (SD)	Hourly Observations ≥35 μg m^−3^	Number of Episodes	Number of Episode Days
Bombay Beach	2992	2.75 (3.30)	0	0	0
Calexico-Ethel Street	3499	12.13 (13.63)	243	77	58
Naval Test Base	2165	5.43 (4.95)	6	4	4
Niland-English Road	3029	8.98 (11.78)	39	16	13
Salton City	2469	4.39 (11.60)	45	16	17
Sonny Bono	2376	4.71 (5.33)	3	3	3

* The total number of possible hourly observations during the study period was 3624.

**Table 2:** Site data summary statistics of concentrations and air pollution episodes for community sites during the study period (1 October 2016 to 28 February 2017).

Site	Hourly Observations	PM_2.5_ Concentration μg m^−3^ Mean (SD)	Hourly Observations ≥35 μg m^−3^	Number of Episodes	Number of Episode Days
On West Hwy 98	2885	9.23 (13.02)	189	67	48
Brawley, on North St	3541	7.08 (10.64)	58	21	17
Brawley, East	2267	3.74 (9.23)	32	11	7
Brawley, High School	2325	6.58 (9.29)	59	16	15
Brawley, West	1492	4.84 (7.61)	11	5	5
Calexico, on Kubler Rd	3468	10.75 (13.54)	287	93	68
Calexico, Alvarez	2724	18.56 (19.62)	530	120	75
Calexico, Encinas Ave and Ethel St	984	10.27 (13.29)	72	18	15
Calexico, Ethel	3224	13.51 (16.24)	379	83	66
Calexico, Housing Authority	3350	12.85 (14.94)	353	100	69
Calexico, Housing Authority West	2910	14.26 (18.91)	423	107	74
Calexico, Residence	2611	10.88 (13.43)	216	55	40
Calipatria, on Hwy 115	3002	4.62 (10.55)	68	24	16
Calipatria, Unified School District	2789	4.44 (12.07)	91	23	18
CCV	3452	8.83 (11.55)	141	40	30
El Centro, Wilson	3489	7.73 (9.87)	117	42	29
Frank Wright	3559	7.6 (11.31)	123	27	19
Heber	2267	8.15 (10.02)	83	30	26
Hidalgo	3461	9.51 (13.46)	193	51	37
Holtville, on Miller Rd	3352	7.99 (10.33)	108	38	31
Holtville, High School	2930	7.89 (11.16)	102	33	24
Holtville, South	3561	8.59 (11.44)	191	43	36
IID, Bombay Beach	801	3.15 (10.99)	14	5	5
IID, Naval Test Base	2219	6.08 (14.28)	50	20	17
IID, Salton City	712	4.98 (13.46)	17	11	12
IID, Salton Sea Park	2868	2.57 (7.90)	33	10	6
IID, Sonny Bono Replicate	574	2.62 (7.60)	6	3	2
IID, Torrez Martinez	1084	8.7 (16.66)	88	16	14
Kennedy	3565	7.84 (10.05)	123	43	32
Meadows	3564	6.45 (9.78)	82	27	22
Mexicali	2486	20.83 (19.44)	571	120	79
Niland	3537	4.34 (8.11)	27	9	9
Ocotillo	1050	4.43 (4.18)	1	1	1
Seeley	3560	8.74 (9.99)	126	56	43
Sonny Bono	1433	27.18 (58.22)	230	21	31
TL Waggoner	2906	4.78 (8.26)	37	15	12
Westmorland, on Lack Road	3555	5.15 (10.43)	47	16	15
Westmorland, Elementary School	803	7.23 (9.81)	42	6	6

**Table 3:** Summary statistics of the number of episode days (i.e., at least one hour during the 24-h period that reached at least 35 μg m^−3^) identified by the government vs. community monitoring.

		Community Monitoring	
		Episode Day	Not an Episode Day
Government Monitoring	Episode Day	74	5
	Not an Episode Day	56	16

## Supplementary Materials

The following are available online at https://www.mdpi.com/1660-4601/16/18/3268/s1, Figure S1: Histograms of PM_2.5_ concentrations observed during the study period at each government monitoring site with red dashed line indicating 35 μg m^−3^, Figure S2: Histogram of the hours in which PM episodes ≥35 μg m^−3^ occurred at the Calexico-Ethel site, Figure S3: Histogram of the day of week in which PM episodes ≥35 μg m^−3^ occurred at the Calexico-Ethel site, Figure S4: Wind roses for the entire study period, and during PM episodes ≥35 μg m^−3^ at each of the government-operated sites, Figure S5: Histograms of PM_2.5_ concentrations observed during the study period at each community monitoring site with red dashed line indicating 35 μg m^−3^.
