# Supplementary material for: Next-Generation Community Air Quality Sensors for Identifying Air Pollution Episodes
Source: Int J Environ Res Public Health. 2019 Sep 5;16(18):3268. doi: 10.3390/ijerph16183268 (PMC6774374; doi:10.3390/ijerph16183268)
Supplement: Supplementary file 1 [file ijerph-16-03268-s001.pdf]

International Journal of
Environmental Research and Public Health

MDPI

Supplementary Information

The calibration model which converts PM sensor counts to mass concentrations is described in our previous paper Carvlin et al., 2017. The model adjusts for relative humidity, which is measured within each monitor. As described in the paper, data used to fit the model were collected from a colocation experiment at one of the government monitoring sites, Calexico-Ethel. The model equation is implemented as a multivariate linear regression as follows:

$$Dylos_{bin1} = \beta_0 + \beta_1 BAM_{PM2.5} + \beta_2 RH + e()$$

in which we used Dylos bin 1 particle counts to relate to BAM $PM_{2.5}$ particle mass concentration because we found to be most associated with $PM_{2.5}$ mass concentrations in the study region. The model adjusts for variations in relative humidity (RH). Although RH can have a non-linear effect on this relationship, generally the RH levels is this region are not high enough for non-linearities to be important. Model coefficients are provided in Carvlin, et al.

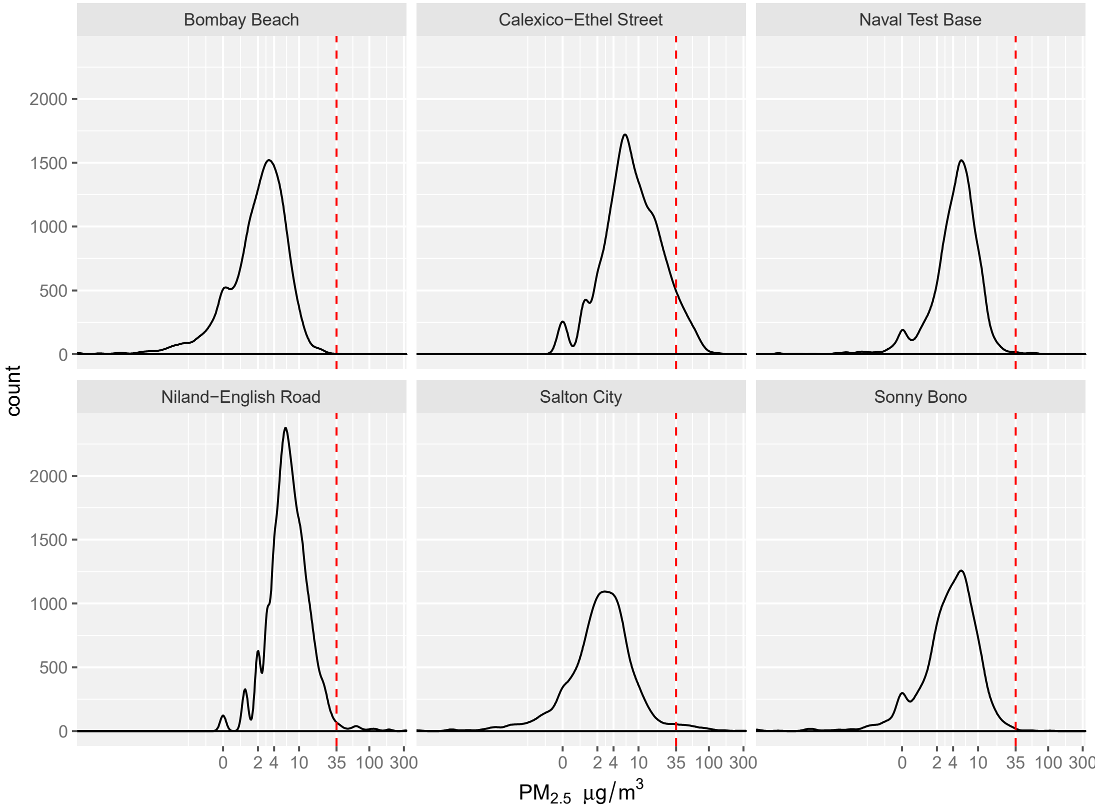

Figure S1. Histograms of $PM_{2.5}$ concentrations observed during the study period at each government monitoring site with red dashed line indicating 35 μg m^{-3}.

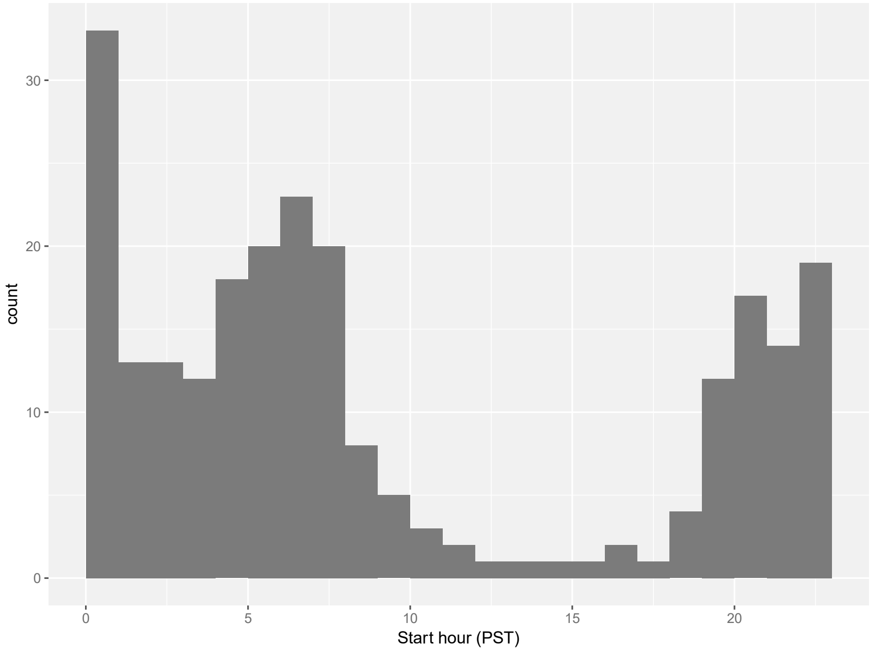

Figure S2. Histogram of the hours in which PM episodes ≥35 μg m^{-3} occurred at the Calexico-Ethel site.

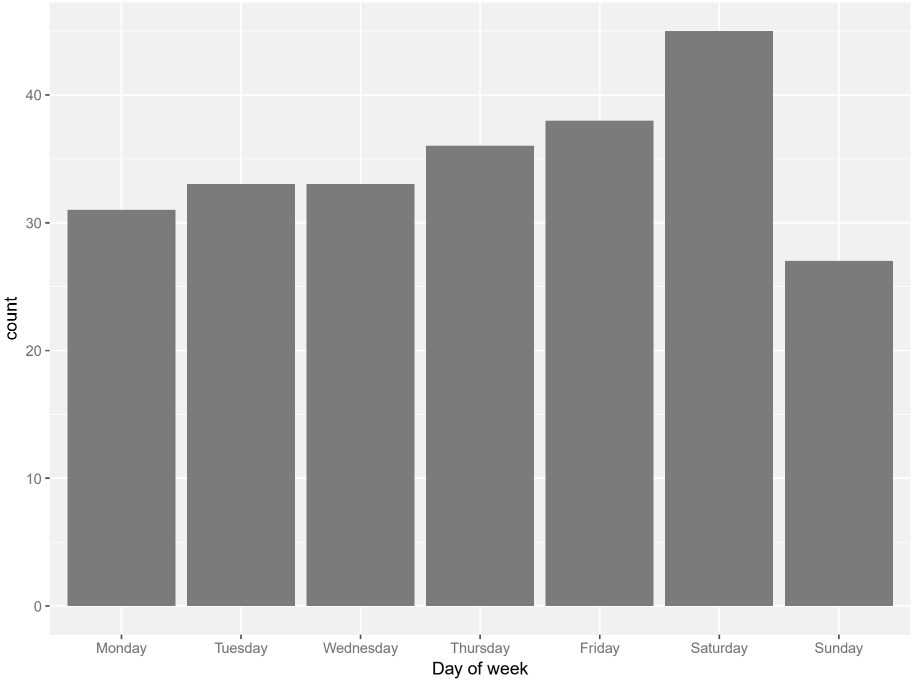

Figure S3. Histogram of the day of week in which PM episodes ≥35 μg m^{-3} occurred at the Calexico-Ethel site.

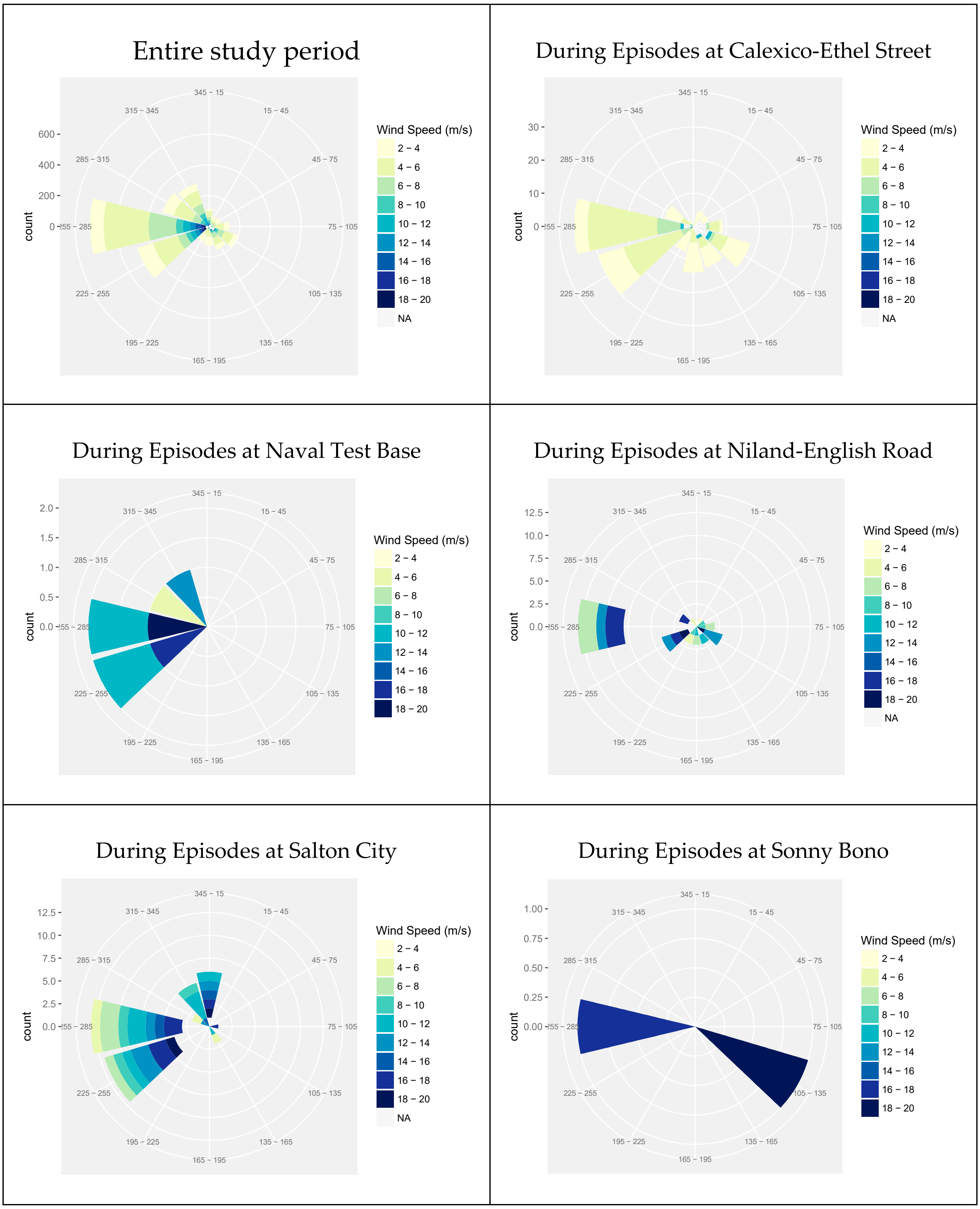

Figure S4. Wind roses for the entire study period, and during PM episodes ≥35 μg m^{-3} at each of the government-operated sites.

International Journal of
Environmental Research and Public Health

MDPI

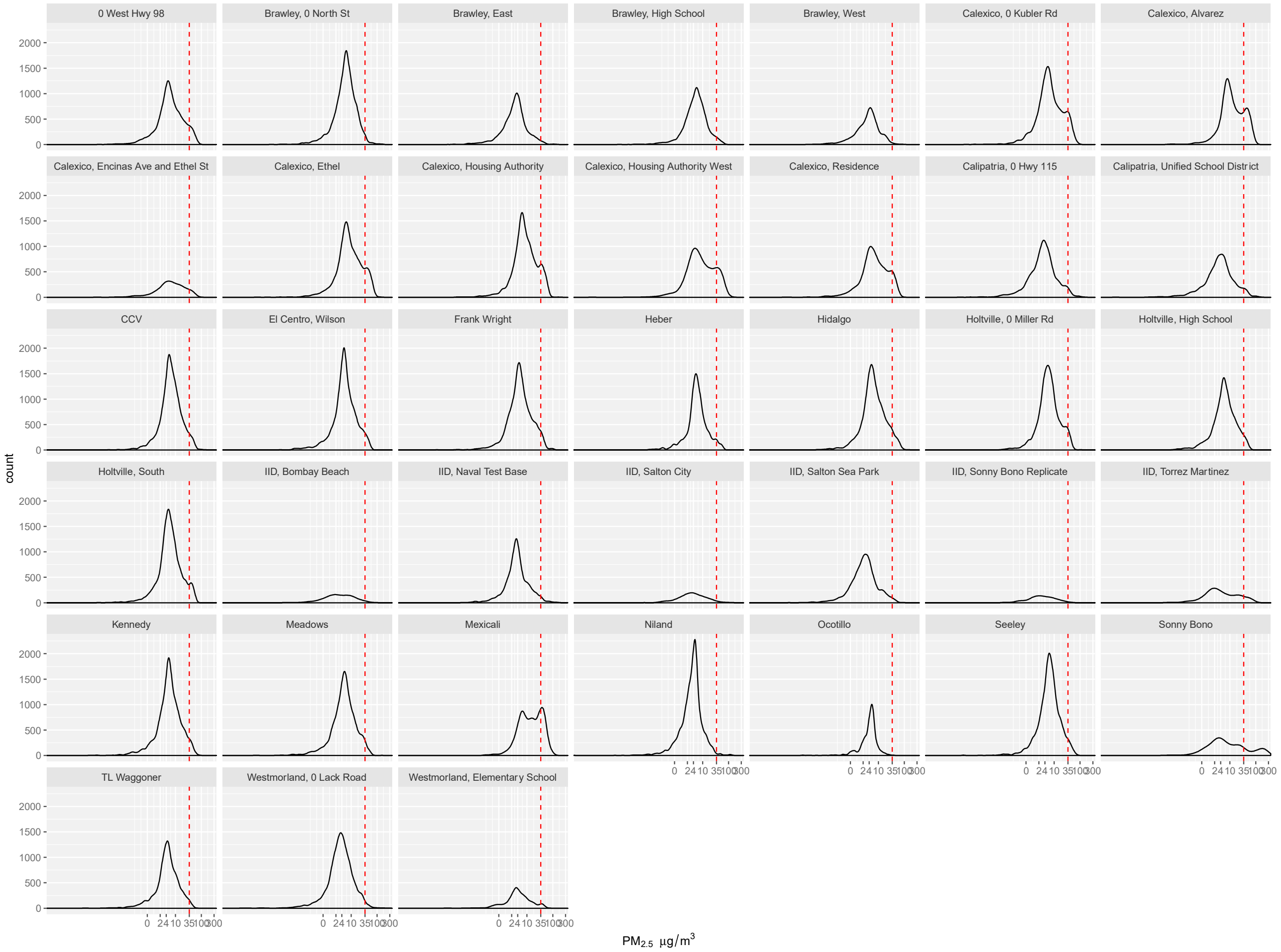

Figure S5. Histograms of $PM_{2.5}$ concentrations observed during the study period at each community monitoring site with red dashed line indicating 35 µg m^{-3}.
